# Reactive Oxygen Species in Crop Plants: Production, Detoxification, Signaling, and Molecular Cross-Talk

**DOI:** 10.3390/plants15142212

**Published:** 2026-07-20

**Authors:** Edappayil Janeeshma, Susmita Das, Sarah Bouzroud, Mohammad Sarraf, Nosheen Akhtar, Hesam Mousavi, Ulkar Ibrahimova, Masayuki Fujita, Mirza Hasanuzzaman

**Affiliations:** 1 Department of Botany, Mes Keveeyam College, Valanchery 676552, India; 2 Agricultural and Ecological Research Unit, Indian Statistical Institute, Kolkata 700108, India; 3 Microbiology and Molecular Biology Team, Center of Plant and Microbial Biotechnology, Biodiversity and Environment, Faculty of Sciences, Mohammed V University in Rabat, Rabat 10000, Morocco; 4 Department of Horticultural Science, Faculty of Agriculture, Shahid Chamran University of Ahvaz, Ahvaz 61357-43311, Iran; 5 Department of Agricultural Sciences, Texas State University, San Marcos, TX 78666, USA; 6 Faculty of Applied Ecology, Agricultural Science, and Biotechnology, University of Inland Norway, 2418 Elverum, Norway; 7 Institute of Molecular Biology Public Legal Entity, Ministry of Science and Education of the Republic of Azerbaijan, Baku AZ 1073, Azerbaijan; 8 Research Institute of Crop Husbandry, Ministry of Agriculture of the Republic of Azerbaijan, Baku AZ 1098, Azerbaijan; 9 Independent Researcher, Mure-cho, Takamatsu 761-0121, Kagawa, Japan; 10 Department of Agronomy, Faculty of Agriculture, Sher-e-Bangla Agricultural University, Dhaka 1207, Bangladesh

**Keywords:** antioxidant system, superoxide, stress signaling, ascorbate–glutathione cycle, H_2_O_2_, plant stress tolerance, cross tolerance, hydrogen peroxide

## Abstract

Although excess generation of reactive oxygen species (ROS) is harmful for plants, at mild concentrations, they are able to positively regulate the metabolic pathway and signaling cascades of the cell. The mechanisms functional under the ROS-induced stress tolerance need to be decoded for the development of new abiotic stress-tolerant crop varieties with a clear vision. This study details ROS generation, roles of enzymatic antioxidants as well as non-enzymatic antioxidants, and ROS-induced cellular events. Emphasis is given to crucial topics such as cyclin-dependent kinases and mitogen-activated protein kinases signaling mechanisms, calcium-mediated cellular cross-talk, and activation or inactivation of various transcription factors. Introduction of different genetic and molecular approaches to manipulate the ROS pathway helps to find out novel recombinant plant varieties. Selection of specific biotechnological tools, appropriate omics analysis and implementation of CRISPR/Cas 9 in crop plants assist the designing of mutants. This review highlights the beneficial roles of oxidative stress, spotlighting the molecular mechanisms that support the physiological, morphological and biochemical modifications of plant cells.

## 1. Introduction

Plants are exposed to various abiotic stressors, such as extreme temperatures, drought, salinity, flooding, metal/metalloid toxicity, anoxia, and hypoxia during different developmental stages of their life cycle, which adversely affect growth, leading to reduced agricultural productivity and financial crisis [1,2]. Exposure to different stress factors results in the accumulation of various reactive oxygen species (ROS) such as singlet oxygen (^1^O_2_), hydrogen peroxide (H_2_O_2_), superoxide (O_2_^•−^), and hydroxyl radicals (OH^•^) due to the imbalance in cellular homeostasis, which leads to oxidative stress [3]. ROS are produced as by-products of metabolic reactions at different cellular compartments, such as chloroplasts, the endoplasmic reticulum, the plasma membrane, mitochondria, and peroxisomes, and they serve a dual role [4]. At lower concentrations, they perform as crucial signaling molecules that facilitate plant resilience; however, under adverse environmental conditions, they accumulate excessively, which acts as a stress factor causing cellular damage. It also results in oxidative damage of major biomolecules, including lipids, nucleic acids, and proteins [5]. Evaluating strategies to detoxify these ROS by inducing enzymatic and non-enzymatic antioxidant defenses at the molecular level is a major focus of current research [6]. Different ROS also perform signaling functions and activate secondary messengers, which lead to sequential expression of specific genes involved in tolerance mechanisms [4].

ROS molecules are produced from various metabolic pathways and they act as an indicator to monitor the status of primary as well as secondary metabolic processes of different cellular compartments [7]. Analysis of the interconnection between plasma membrane fluctuations, ROS production and cytoplasmic calcium (Ca^2+^) content is a key step in detecting and mitigating the impact of environmental changes on plants [8]. Thus, a literature survey on the functional synchronization of ROS with different hormones, reactive nitrogen species (RNS), mitogen-activated protein kinases (MAPKs), and transcription factors (TFs) is essential for crop improvement [9].

Manipulating ROS pathways for the local regulation of cell-to-cell communication can serve as a strategy to facilitate crop improvement [10]. The induction and manipulation of genes encoding antioxidant activities help regulate ROS synthesis, scavenging, and reductive repair [11]. Therefore, a comprehensive approach to ROS-associated pathways is essential for designing future crops by modifying ROS generation and detoxification [12].

Over the past two decades, ROS have been established as double-edged molecules— being toxic at high concentrations but indispensable as signaling entities that mediate stress perception, developmental transitions, and cross-tolerance to multiple stresses [13]. Several excellent reviews have described the general mechanisms of ROS production and scavenging, antioxidant systems, and MAPK or hormonal signaling under abiotic stress [14]. However, most of these works have primarily focused on model species (such as *Arabidopsis thaliana thaliana*, *Nicotiana tabacum*) or general cellular processes, often overlooking species-specific and organ-specific ROS regulation in economically important crops [14]. In recent years, a paradigm shift has occurred in understanding ROS signaling as a spatiotemporally dynamic and compartmentalized network rather than a simple linear response [15]. Despite numerous studies, several questions remain unresolved. How do ROS signatures differ among organelles and across crop species with distinct metabolic architectures? What regulatory networks integrate ROS with hormone signaling (ABA, ethylene, auxin, gibberellins) and ion fluxes under combined or sequential stresses? And how can this mechanistic understanding be translated into breeding or genome editing strategies to enhance redox resilience in crops? To address these questions, the present review gives a comprehensive, crop-oriented synthesis of ROS biology under abiotic stress, integrating production sites, detoxification pathways, and signaling cross-talk at the cellular and organ-specific levels.

Unlike most previous reviews that treat ROS functions as isolated events within single organelles or generalized model systems, this review introduces a multi-organ, crop-specific framework for understanding ROS biology. It integrates spatial (organellar and tissue-level) and temporal (developmental and stress-phase-specific) dimensions of ROS dynamics to reveal how localized redox events coordinate systemic responses in crops. The manuscript also synthesizes recent mechanistic breakthroughs in ROS perception, redox post-translational modifications, and inter-organellar communication, framing them within a translational perspective that connects molecular redox signaling to field-level crop performance. This integrative model provides greater conceptual depth and applied relevance, distinguishing the present work from earlier descriptive reviews and offering new directions for future redox-based crop improvement.

## 2. Production of ROS in Crop Plants

### 2.1. Types of ROS and Their Sources Within Plant Cells

ROS are oxygen derivatives that exhibit higher reactivity than molecular oxygen (O_2_) itself [16,17]. Each type of ROS possesses unique chemical properties. For instance, ^1^O_2_ can oxidize lipids, proteins, and DNA (mainly guanine residues) [16,18,19]; O_2_^•−^, similar to ^1^O_2_, has a half-life of 1–4 µs and reacts with Fe-S proteins; and hydroxyl radicals (OH^•^) are highly reactive and unstable, with a half-life of just 1 nano second [16,19]. In contrast, H_2_O_2_ is relatively stable, with a half-life exceeding 1 milli second, making it a key ROS in cellular signaling. Regulation of intracellular ROS level by the interaction of ROS generated in different cellular compartments is collectively known as “ROS processing systems” [18]. H_2_O_2_ is scavenged by different biocatalyst, particularly catalases (CATs) and ascorbate peroxidases (APXs), which are crucial for H_2_O_2_ metabolism. Other enzymes functional in the detoxification of peroxides include glutathione *S*-transferases (GSTs) [20] and peroxiredoxins (PRXs) [21], such as glutathione peroxidases (GPXs). Despite their name, GPXs are now understood to function as thioredoxin (TRX)-dependent PRXs [22]. The detoxification reaction depends on the regeneration of reductants like ascorbate, glutathione (GSH), and TRX, ultimately relying on NADPH. O_2_^•−^ is primarily processed by superoxide dismutase (SOD), whereas OH^•^ and ^1^O_2_ are scavenged mainly through non-enzymatic reactions [23,24].

**Figure 1:**
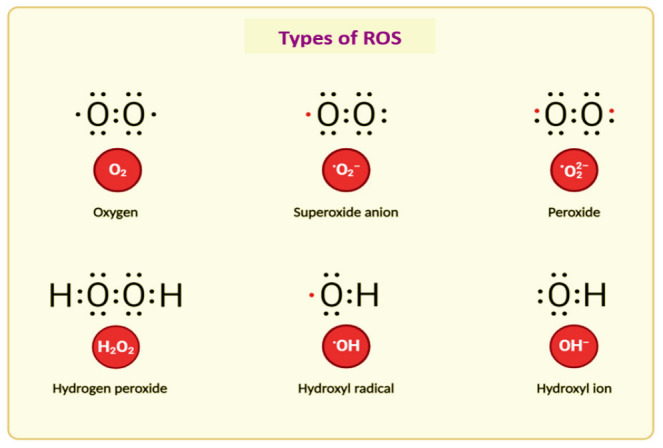
Types of ROS in plants.

ROS in plant cells arise from various intracellular sources and include both free radicals and non-radicals [25]. Among the free radical ROS, O_2_^•−^ is generated primarily in mitochondria, chloroplasts, plasma membrane NADPH oxidases (NOXs), and peroxisomes due to leakage from electron transport chains and by the activity of different enzymes (Figure 1).

Cytochromes are multimeric electron carrier biomolecules that facilitate the reduction of O_2_ to O_2_^•−^ via NADPH-dependent oxidases. Cytochrome-mediated detoxification is prominently observed at the plasma membrane. Alternatively, ROS are produced in the apoplast through the activity of pH-dependent peroxidase (POD), oxalate oxidase, and amine oxidase [26,27]. ROS are known to induce a range of structural and physiological responses as well as the degradation of macromolecules in plants [28,29].

### 2.2. Role of Chloroplasts, Mitochondria, and Peroxisomes in ROS Production

Organelles involved in highly oxidative metabolic activities or intense electron flow, like chloroplasts, mitochondria, and peroxisomes, are significant sources of free radicals in plants. ROS are produced by PODs and amine oxidases in the cell walls and by NOX in the plasma membrane, often in response to stress signals [30]. Within the chloroplast, oxygen is consistently generated through photosynthetic electron transport and is simultaneously reduced and assimilated.

The chloroplast contains an intricately organized membrane system that acts as the major site of photophosphorylation [31]. The core components of this system, the photosystems I (PSI) and II (PSII), are the primary sources of ROS generation. Chloroplasts contribute more significantly to ROS generation as compared to mitochondria. The interaction between photosynthetic pigments and light, specifically the triplet chlorophyll state and the ETC, represents a major source of free radicals [4]. Abiotic stress factors such as drought, salinity, temperature extremes, and excess light can lead to water stress and reduced CO_2_ concentrations, resulting in the formation of O_2_^•−^at the PSs via the Mehler reaction. This O_2_^•−^ is quickly transformed into H_2_O_2_ with the support of membrane-bound Cu/Zn SOD at PSI [32]. The leakage of electrons from the electron transport chain (ETC) of PSI, particularly from the 2Fe-2S and 4Fe-4S clusters, also contributes to ROS formation [33].

QA and QB, the electron acceptors of PSII, are considered as the major sites of electron leakage, leading to the generation of O_2_^•−^ radicals. These O_2_^•−^ radicals are subsequently converted into highly toxic OH^•^, through the H_2_O_2_ intermediate via the Fenton reaction [33]. PSII is also responsible for generating 1O_2_ in two mechanisms. First, abiotic stress disrupts the balance between light harvesting and energy utilization, resulting in the formation of triplet chlorophyll (3Chl**), which reacts with dioxygen (^3^O_2_) to produce ^1^O_2_ [2]. Second, when electron transport is severely diminished at PSII, ^1^O_2_ is generated by the light-harvesting complex (LHC) [34]. Moreover, this accumulated ^1^O_2_ in the chloroplast leads to peroxidation of membrane lipids (particularly polyunsaturated fatty acid, PUFAs), damage of membrane proteins, and impairment of the P680 reaction center of PSII [35,36,37,38].

Mitochondria, while crucial for cellular energy production, are also an important source of ROS like H_2_O_2_ and O_2_^•−^ chloroplasts [39]. The mitochondrial electron transport chain (mtETC) generates ROS molecules, as it contains highly energized electrons capable of reducing O_2_ to form ROS. Complexes I and III of the mtETC are the primary contributors to ROS production [40,41].

Complex I (NADH dehydrogenase) transfers electrons to O_2_ and forms O_2_^•−^ within its flavoprotein domain. The generation of ROS is enhanced during reverse electron flow from Complex III to Complex I, driven by a lack of NAD^+^-linked substrates and controlled by ATP hydrolysis [42]. Complex III (ubiquinone–cytochrome c oxidoreductase) transfers electrons to cytochrome c1, resulting in the formation of ubisemiquinone radical. This compound leaks electrons to O_2_, generating O_2_^•−^ [43]. In addition to these complexes, various mitochondrial matrix enzymes also contribute to ROS generation. Under abiotic stress, mitochondria accumulate higher levels of ROS compared to optimal conditions [44]. This is due to stress-induced disruption of the coupling between ETC and ATP synthesis, resulting in over-reduction in the ubiquinone (UQ) pool and subsequent ROS generation [45].

Peroxisome is a single-membrane-bound spherical microbody that serves as a key site for intracellular H_2_O_2_ development due to its specific roles in oxidative metabolism [46]. In addition to H_2_O_2_, peroxisomes generate O_2_^•−^ from different metabolic events. In the first event, O_2_^•−^ generates as a by-product when xanthine oxidase catalyzes uric acid formation. At the second event, ETC located in the peroxisomal membrane releases O_2_^•−^ into the cytosol, which is dependent on NADPH and composed of NADH and cytochrome *b*. Additionally, peroxisomal membrane polypeptides (PMPs) also contribute to O_2_^•−^production.

Non-optimal conditions, such as limited water availability and closed stomata, decrease in CO_2_ to O_2_ ratio, leading to a high rate of photorespiration and glycolate formation. By the action of glycolate oxidase in the peroxisome, H_2_O_2_ gets released [47,48,49]. Other metabolic processes that contribute to peroxisomal ROS production include β-oxidation of fatty acids and the flavin oxidase-mediated reactions [50,51].

### 2.3. Role of Polyamines and Polyamine Oxidases in ROS Generation

Polyamines are polycationic molecules that contain two or more amino groups (–NH_3_^+^) universally found in eukaryotic and prokaryotic cells. Small, positively charged biomolecules such as putrescine (Put), spermidine (Spd), and spermine (Spm) are the major PAs in plants [52]. Polyamines are synthesized from arginine, ornithine, proline, and methionine [53]. The indirect involvement of polyamines in certain biotic and abiotic stress responses and associated production of ROS are discussed here [54]. Arginine decarboxylase (ADC), ornithine decarboxylase (ODC) spermidine synthase (SPDS), thermospermine synthase (ACL5), ornithine/lysine decarboxylases (O/LDCs), and spermine synthase (SPMS) are the prominent enzymes involved in the biosynthesis of PAs. Stress tolerance, cell division, organ development, senescence, and fruit ripening are the major processes regulated by PAs. Polyamines promote the generation of H_2_O_2_ and nitric oxide (NO) and their homeostasis regulates the ROS level through several cellular events. The signaling role of apoplastic PA-derived H_2_O_2_ under abiotic stress was demonstrated using a series of tobacco (*Nicotiana tabacum* cv Xanthi) mutants overexpressing/downregulating apoplastic polyamine oxidase (PAO) [55]. The balanced regulation of polyamines and ROS is crucial for plant adaptive responses and survival under saline conditions [56]. A PAO is a catalytic convertor that oxidizes PAs at secondary amino groups, transforming them into other products. This process is essential to regulating cellular PA concentration as well as ROS production. This enzymatic flavoprotein uses molecular oxygen as an acceptor and oxidizes a carbon–nitrogen bond in a secondary amino group of PAs. Three major reactants of PAO-mediated reactions are water, oxygen, and a polyamine with primary as well as secondary amino groups. H_2_O_2_, an amino-aldehyde, and a primary amine are the major products of the reaction [57].

Polyamine oxidases (PAOs) are involved in the catabolism of PA. Plant PAOs are classified into two major groups. One group is responsible for the terminal catabolism of PAs and the other group catalyzes back-conversion reactions of PAs accompanied by the generation of H_2_O_2_. In maize and tobacco, the PAO and the generated ROS are directly related to wound healing [54].

### 2.4. Role of NADPH Oxidase in ROS Generation

The NADPH oxidases or respiratory burst oxidase homologs (RBOHs) are biocatalysts involved in the generation of ROS by scavenging O_2_^•−^**,** which is rapidly converted into H_2_O_2_ [58]. These enzymes are involved in the transfer of electrons from cytosolic NADPH or NADH to apoplastic O_2_ and generate O_2_^•−^. This O_2_^•−^ is further converted to H_2_O_2_ by the action of SOD. NOXs have a crucial role in plant development and stress tolerance. RBOH (A–J) enzymes have been isolated and identified from *A. thaliana*, confirming this role. The major functions of RBOHD and RBOHF have been identified as contributing to abiotic stress tolerance in plants [54]. Exogenous application of PAs (viz. putrescine, spermidine and Spm) stimulates the NOX-PM H^+^-ATPase cascade, resulting in augmented production of O_2_^•−^ and H_2_O_2_. NOX and PM H^+^-ATPase activities and PAO activities have a strong direct correlation in plants. Correlation of the present observations with earlier findings point out that H_2_O_2_ and Ca^+ 2^ interact with the NOX-PM H^+^-ATPase cascade. H_2_O_2_ and Ca^2+ ^ are also involved in PA metabolism during early root growth in *Vigna radiata* seedlings [59].

## 3. ROS Production and Oxidative Stress Under Various Abiotic Stresses

H_2_O_2_ accumulates in cells during exposure to various abiotic stresses such as drought, salinity, heavy metal exposure, extreme temperatures, and UV radiation, which cause oxidative damage [60,61]. Abiotic stresses disrupt cellular homeostasis and metabolic activities, resulting in the accumulation of ROS through multiple biochemical pathways.

One primary source of ROS under non-optimal conditions is the chloroplast, particularly during photosynthesis [62]. Under elevated light intensity or when the electron transport chain is over-reduced, electrons can leak from PS I and II complexes, leading to the generation of O_2_^•−^ radicals [4,63]. Additionally, photorespiration, which is enhanced under high light and low CO_2_ conditions, contributes to H_2_O_2_ production in peroxisomes [63,64,65,66].

Mitochondria are another key site for ROS generation, especially during respiration. Under abiotic stress, the electron transport chain in mitochondria can become disrupted, causing electron leakage and the formation of O_2_^•−^ radicals [41]. This is particularly pronounced when there is an imbalance between the production of ATP and the electron flow through the respiratory chain. Peroxisomes play a significant function in ROS production during abiotic stress through various metabolic processes such as fatty acid β-oxidation, photorespiration, and the detoxification of reactive aldehydes. The activity of oxidases in peroxisomes can lead to the generation of H_2_O_2_, which, if not scavenged efficiently, can contribute to oxidative stress [4].

Another important source of ROS under abiotic stress is the plasma membrane-bound NOX, also known as respiratory burst oxidase. This enzyme complex is activated in response to various stress signals, leading to the production of O_2_^•−^ radicals. These radicals can subsequently dismutate to form H_2_O_2_, which acts as a signaling molecule but can also cause oxidative damage if it accumulates in excess [67]. Heavy metals and metalloids such as cadmium, mercury, and arsenic can induce ROS production by interfering with antioxidant defense mechanisms and disrupting redox homeostasis. These metals can directly generate ROS through Fenton and Haber–Weiss reactions, where transition metals catalyze the conversion of H_2_O_2_ into highly reactive OH^•^ radicals [68,69].

Abiotic stresses often impair the balance between ROS production and the antioxidant defense system, which includes enzymes like SOD, CAT, and various PODs, as well as non-enzymatic antioxidants like ascorbate, GSH, and flavonoids. The diminished capacity of these defenses under stress conditions leads to the accumulation of ROS and the onset of oxidative stress, resulting in cellular damage, lipid peroxidation, protein oxidation, and DNA damage [4,70]. The details of the generation of ROS under specific abiotic stress conditions are discussed in the next section.

### 3.1. Drought

A plant’s response to drought depends on its inherent strategy as well as the duration and severity of the drought. Prolonged drought stress can lead to oxidative damage due to the overproduction of ROS [71].

Through abscisic acid (ABA)-induced signal transduction, plants stimulate closure of stomata that aid in controlling water loss by reducing transpiration. At the same time, it limits CO_2_ intake, which affects net photosynthesis and increases ROS production under drought stress [72,73]. Drought-induced modulations in leaf anatomy and Glaucousness synthesis is another stress-tolerant adaptation [74,75,76].

Inhibition of chlorophyll biosynthesis, restructuring of thylakoids, and formation of antenna-depleted PS II, as well as remodulation in the shapes of chloroplast and vacuole, result in the reduction in photosynthetic and respiratory activities [77,78]. This results in a significant decline in chlorophyll *a*, chlorophyll *b*, and total chlorophyll content in plants such as *Gossypium hirsutum* [79], *Helianthus annuus* [80], and *Catharanthus roseus* [81]. Water deficit progressively suppresses photosynthesis by disrupting thylakoid electron transport, C_3_ cycle, and stomatal control of CO_2_ supply and accumulation [76,82]. Severe drought limits photosynthesis by reducing the activity of ribulose-1,5-bisphosphate carboxylase (Rubisco), accompanied by decreased rates of carboxylation, Rubisco regeneration, stromal fructose bisphosphatase activity, and quantum efficiency of PS II [83].

Drought stress also reduces chlorophyll-binding proteins, diminishing the light-harvesting pigment protein complex associated with PS II [75,84,85]. This decline is mainly due to chloroplast damage caused by ROS induced by drought [78]. Conversely, drought increases xanthophyll pigments such as zeaxanthin and antheraxanthin, which protect plants under stress by participating in the xanthophyll cycle that inhibits ROS production [86,87].

Drought negatively impacts photosynthetic enzymes like NADP-dependent glyceraldehyde 3-phosphate dehydrogenase, phosphoenolpyruvate carboxylase, NAD-dependent malate dehydrogenase, phosphoribulose kinase, fructose-1,6-bisphosphatase, and sucrose phosphate synthase [88]. The photosynthetic electron transport chain activity is finely tuned to CO_2_ availability in the chloroplast and often declines in PS II activity under drought [89,90]. Additionally, drought disrupts both cyclic and non-cyclic electron transport during the light reactions of photosynthesis [91].

Drought stress enhances ROS production by limiting CO_2_ fixation, reducing NADP^+^ regeneration, and causing an over-reduction in the photosynthetic electron transport chain. For instance, in drought-stressed wheat, photosynthetic electron leakage to the Mehler reaction increased by about 50% [92]. Similar increases were observed in sunflowers. Both the Mehler reaction and enhanced photorespiration contribute to ROS generation under drought, with photorespiration likely accounting for over 70% of total H_2_O_2_ production. The chloroplast, although robust against ROS due to scavenging enzymes and metabolites, faces threats under drought stress. The hydroxyl radical, produced in the thylakoids through iron-catalyzed reduction in H_2_O_2_, is particularly damaging. This radical has a short half-life but a strong oxidizing potential, reacting with almost every biological molecule. No known enzymatic reaction can eliminate the hydroxyl radical, leading to damage to thylakoid membranes and the photosynthetic apparatus. Studies in *Salvia officinalis* indicated that the singlet oxygen is the major ROS involved in the degradation of the photosynthetic apparatus, not H_2_O_2_, as evidenced by the strong degradation of β-carotene and α-tocopherol [14]. Changes in the protein pool of chloroplasts introduce modifications in leaf gas exchange properties andit leads overproduction of ROS molecules [93].

### 3.2. Salinity

Excessive Na^+^ and Cl^−^ influx parallel with K^+^ efflux results in ion imbalances and K^+^ deficiency due to impaired root membrane selectivity due to excess salt concentration [94]. Salinity stress also triggers the accumulation of ROS like ^1^O_2_, O_2_^−^, and H_2_O_2_, which cause oxidative damage to primary metabolites at high concentrations [95]. This stress increases ROS production and signaling, affecting the plant’s phenotype.

Stomatal conductance is reduced under salinity stress, limiting CO_2_ supply and increasing photorespiration in C_3_ plants, which further produces ROS, primarily H_2_O_2_. Na^+^ and Cl^−^ hyperaccumulation in the cytosol disrupts electron transport in photosystems (PS) by preventing the activity of electron acceptors in PS I and PS II [96]. Molecular oxygen, receiving these electrons, leads to the generation of O_2_^•−^ and other ROS [97]. The primary sites for ROS synthesis under abiotic stress are chloroplasts [98], mitochondria [99], and peroxisomes [100].

In chloroplasts, salinity stress enhances ROS synthesis in PSI and PSII during illuminated conditions. Mitochondrial electron transport chain Complexes I and III are major sources of salinity stress-induced ROS. Imbalanced reduction in the ubiquinone pool under salinity stress can lead to electron leakage into molecular oxygen from Complexes I and III, producing O_2_^−^ radicals [32]. Oxidative stress from high salinity can increase H_2_O_2_ production during photorespiration, fatty acid β-oxidation, and imbalance of O_2_^−^ in plant cells [101,102]. Additionally, cell wall-associated POD in the apoplastic region generates O_2_^•−^ by oxidizing NADPH and transferring electrons to molecular oxygen [103].

High cytosolic Ca^2+^ is essential for activating stress signaling pathways in response to salinity. This elevation is regulated by three ion channels: depolarization-activated Ca^2+^-permeable channels, hyperpolarization-activated Ca^2+^-permeable channels, and voltage-independent Ca^2+^-permeable channels (non-selective cation channels). Components including cyclic nucleotide-gated channels (CNGCs), annexin, glutamate receptors, and mechanosensitive channels were involved in the regulation of these three ion channels [104].

Prolonged elevation of cytosolic Ca^2+^can be detrimental, leading to ROS production and reduced plant growth. To mitigate this, plants have developed mechanisms like the Ca^2+^ efflux system and a complex self-amplification mechanism to balance basal cytosolic Ca^2+^ levels and minimize ROS production. Signal transduction from cell to cell is also vital in this context. The “ROS-Ca^2+^ hub” is a self-amplification mechanism where excessive ROS production due to salinity stress leads to Ca^2+^ influx via ROS-activated Ca^2+^ channels, which in turn activate NOX to produce more ROS [105,106]. This process amplifies weak signals into fast ROS-Ca^2+^-mediated signals, and is upregulated by the activation of HACC, glutamate receptors, and NOX phosphorylation at the plasma membrane [107].

The ROS-Ca^2+^ hub plays several key roles, including hormonal signal transduction (HST), osmotic modification, mineral nutrition, cell elongation, and programmed cell death. HST is crucial for early stress signaling cascades, regulated by the ROS-Ca^2+^ hub and involving hormones such as ABA, ethylene (ET), brassinolide, auxins, methyl jasmonate, polyamines, and salicylic acid (SA) [108,109,110,111,112,113,114]. This mechanism can overlap with hormone signaling to initiate stress responses in plants [115].

The ROS-Ca^2+^ hub was first identified in root elongation zones and later in pollen tubes [115]. It regulates cytosolic free Ca^2+^ levels in the elongation zone, promoting the formation of cytoskeleton bundles and exocytosis [116]. Activation of CNGC genes and respiratory burst oxidase (RbohC) also contributes to the ROS-Ca^2+^ hub in *A. thalian* [117]. This hub involves *A. thaliana CNGC18*, *GLR1.2*, *GLR3.7*, *RbohH*, and *RbohJ*, particularly in elongating pollen tubes [117,118,119,120]. Future research is needed to explore the molecular basis of ROS-Ca^2+^ hub-mediated physiological processes in plants.

Cytosolic potassium (K^+^) homeostasis is critical for salinity stress tolerance. K^+^ leakage under saline conditions activates caspase-like proteases and endonucleases, leading to programmed cell death [121,122]. While genetic engineering and foliar applications have attempted to reduce K^+^ loss, recent evidence suggests that K^+^ efflux plays a significant signaling role in regulating plant growth and development under stress. Soil salinity induces K^+^ leakage through K^+^ selective depolarization-activated outward rectifying channels (KORK or GORK in *A. taliana*) [123]. These GORK channels, part of the Shaker family of transporters, are highly voltage-sensitive and respond to membrane depolarization under salinity or ROS stress [124].

### 3.3. Extreme Temperatures

High temperature stress disrupts membrane fluidity, increasing cytosolic Ca^2+^ levels and initiating Ca^2+^ signaling through plasma membrane-localized cyclic nucleotide-gated channel (CNGC) family proteins and several Ca^2+^ sensors like calmodulins (CaMs), CDPKs, CMLs (CaM-like proteins), calcineurin B-like proteins (CBLs), and Ca^2+^ binding proteins [125]. The Ca^2+^/CaM-binding protein kinase (CBK3) targets *HSFA1a* during heat stress responses in *A. thaliana* [126]. Concurrently, heat-induced Ca^2+^ signaling is mediated by respiratory burst oxidase homolog (RBOH) proteins, initiating an ROS burst in the apoplast. ROS generated by RBOHs are transported into the cell via aquaporins, triggering further Ca^2+^ release by TPC1 channels to regulate various signaling pathways [127,128]. The interaction between Ca^2+^-ROS signaling with other hormone signaling components activates several downstream pathways, regulating heat shock transcription factors (HSFs) and inducing heat stress responses [129].

Temperature changes sensed by plant receptors initiate complex signaling networks, leading to a reduction in membrane thermostability, higher malondialdehyde (MDA) accumulation, and the generation of ROS such as ^1^O_2_, H_2_O_2_, O_2_^−^, etc. In plants, ROS are primarily produced by RBOH proteins, plasma membrane-localized NOX, and various PODs, which are regulated by the ROS gene network [130]. These ROS are decoded by sensors like GPXs, receptor-like kinases (RLKs), Cys-rich receptor-like kinases (CRKs), serine/threonine protein kinase (OXI1), and redox responsive TFs, initiating stress-specific signals for gene expression and protein synthesis [131,132,133].

Uncontrolled ROS overproduction ultimately leads to an oxidative burst, disruption of proteins, biomolecules, and membranes, thereby affecting photosynthesis and leaf physiology [134,135,136]. For instance, *Cicer arietinum* and *Oryza sativa* L. plants exposed to heat stress (32/20 °C Day/Night, 7 days) exhibited a 6.5-fold increase in H_2_O_2_ accumulation, resulting in oxidative damage compared to controls [137,138]. Nonetheless, studies have demonstrated the importance of ROS and its regulatory systems at various stages of plant development in response to heat stress [139]. Heat stress has been shown to induce the expression of heat shock proteins HSP17.6 and HSP18.2 in *A. thaliana* cell cultures, with ROS inhibitors reducing HSP gene expression, suggesting that H_2_O_2_ is essential for efficient heat shock gene expression [140].

Recent studies have evidenced the potential of miR398 (miRNAs) to down-regulate the activity of antioxidant enzymes by the influence of heat response TFs. This results in the accumulation of ROS and induces heat stress responses via changing *HSFA1* functions [141,142]. In *O. sativa*, the NAC TFs gene (*SNAC3*) modulates H_2_O_2_ homeostasis by controlling ROS-associated genes, acting as a positive regulator under high temperatures [143]. Furthermore, studies on ROS biosynthesis mutants, such as NADPH oxidase (*atrbohB* and *atrbohD*), indicate that maintaining optimal ROS levels is essential for thermotolerance [144].

### 3.4. Metal/Metalloid Toxicity

Toxic metals and metalloids are highly reactive and induce toxicity in plant cells through various mechanisms depending on their type, nature, and concentration. Initially, these toxic elements generate excessive ROS [145,146,147,148]. This toxicity disrupts numerous crucial physiological and molecular functions in plants [149,150]. They deactivate or suppress various enzymes, denature proteins, and compromise cell membrane integrity, thereby hindering metabolism, photosynthesis, and respiration while promoting ROS and free radical production. Elevated ROS levels oxidize important metabolites involved in the normal cellular events [151]. Additionally, methylglyoxal (MG), a reactive oxidative compound formed under abiotic stresses including metal/metalloid toxicity, damages cellular ultrastructure, causes mutations, and can lead to cell death [152].

Plants possess an antioxidant defense system and glyoxalase systems to counteract excessive ROS and MG. Non-enzymatic components (like ascorbic acid (AsA), GSH, phenolic compounds, alkaloids, α-tocopherol, and non-protein amino acids) and enzymatic components (such as SOD, CAT, APX, glutathione reductase (GR), monodehydroascorbate reductase (MDHAR), dehydroascorbate reductase (DHAR), GPX, and GST) form the antioxidant defense system [151,153].

The glyoxalase system detoxifies MG through two key enzymes, glyoxalase I and glyoxalase II [154]. Besides these systems, plants synthesize osmolytes (proline, glycine betaine, trehalose) and chelating agents (phytochelatins, metallothioneins, proteinogenic amino acids, non-proteinogenic amino acids, and anthocyanins) to mitigate stress [155]. The effectiveness of these defense systems against ROS and MG, along with improved synthesis of osmolytes and chelating agents, determines a plant’s stress tolerance. This efficiency varies significantly among plant genotypes and stress intensities. Higher concentrations of toxic metals/metalloids reduce the survival capacity of plants [156].

Under metal/metalloid toxicity, ROS production increases dramatically, disrupting the balance between ROS and antioxidant enzymes [151,156,157]. Under optimal conditions, ROS play vital roles in regulating gene expression and controlling processes like the cell cycle, growth, stress responses, signaling, programmed cell death, pathogen defense, and development. The imbalance in oxygen activation or reduction during metabolic activities under stress leads to excessive ROS production in various cellular compartments, including plastids, peroxisomes, mitochondria, cytosol, and apoplast, with chloroplasts being the primary site [30].

Toxic metals are classified as redox-active [e.g., iron (Fe), copper (Cu), chromium (Cr), cobalt (Co)] and non-redox-active [e.g., cadmium (Cd), lead (Pb), zinc (Zn), nickel (Ni), aluminum (Al)]. Redox-active metals can directly generate ROS through Haber–Weiss and Fenton reactions. Non-redox-active metals enhance ROS production by depleting the antioxidant GSH pool, inducing NOX, displacing essential cations from enzyme binding sites, activating calcium-dependent systems, and influencing iron-mediated processes [145,146,147].

Excess ROS production under metal/metalloid stress has been documented in numerous studies. Under the exposure of Cd toxicity, the rapeseed seedlings showed a significant increase in H_2_O_2_ content [158]. Similar increases in ROS were observed in *O. sativa*, mustard, mung bean, and luffa under various metal stresses [159,160,161,162]. High ROS levels cause lower ATP production, higher lipid peroxidation, membrane impairment, and DNA damage under lead stress, with similar effects seen with other metals like Zn, Cu, Ni, manganese (Mn), Al and boron (B) [163,164,165,166,167,168].

### 3.5. Flooding

Oxygen availability for plants can range from normal levels (normoxia) to partial oxygen deficiency (hypoxia) and total oxygen deficiency (anoxia during flooding) on flooding [169]. During hypoxia, the reduced oxygen partial pressure restricts ATP production through oxidative phosphorylation in the mitochondria [170]. Oxygen deficiency leads to a significant reduction in ATP production because molecular oxygen, a crucial terminal electron acceptor, is unavailable [171].

Under normal oxygen levels, oxidative phosphorylation can produce up to 38 ATP molecules from one glucose molecule. In the absence of oxygen, anaerobic processes such as fermentation and glycolysis can generate only 4–8 ATP molecules. This limited ATP production involves enzymes like pyrophosphate-dependent phosphofructokinase and UDP-dependent sucrose synthase, which operate in the cytosol, unlike in plastids, where pyrophosphate-dependent enzymes are absent, yielding only two ATP molecules from carbohydrate oxidation [160]. Thus, glycolysis coupled with lactic and alcoholic fermentation becomes the primary ATP source, albeit inefficiently. This inefficiency directs to the overproduction of toxic by-products like ethanol and lactate, with lactate also causing cytosol acidification.

Oxygen deficiency impairs electron flow in the mitochondrial electron transport chain (ETC), initiating oxidative damage that worsens upon reoxygenation. Prolonged oxygen deprivation results in an energy crisis, cytoplasmic acidosis, and the build-up of harmful metabolites, including acetaldehyde. It also diminishes overall protein synthesis and increases the development of reactive oxygen and nitrogen species, which in turn trigger lipid peroxidation. During anaerobic conditions, glycolysis and both alcoholic and lactic acid fermentation are enhanced. The rapid activation of lactate dehydrogenase, observed in many plants, initially acidifies the cytoplasm, followed by a shift towards predominantly alcohol fermentation over time [171].

Changes in oxygen levels significantly impact mitochondrial respiration, affecting mitochondrial metabolism, the proton motive force, ATP/ADP ratios, the antioxidant pool, the NAD(P)H redox state, and reactive oxygen/nitrogen species (ROS/RNS) homeostasis, potentially initiating mitochondrial signaling [172]. Wagner et al. [173] identified various signaling pathways in mitochondrial retrograde responses. For example, treatment with antimycin-A in *A. thaliana* L. inhibited Complex III, triggering the overexpression of hypoxia-responsive genes, increased ROS, and disrupted ATP/ADP ratios. Mitochondrial signaling also up-regulates Uncoupling Protein (UCP1), which reduces ROS production to enhance hypoxia tolerance [174]. Respiratory burst oxidase homolog D (RBOHD) is a key hypoxia gene in *A. thaliana*, and its transient expression reduces ROS outbursts during hypoxia, aiding seedling survival through hypoxic acclimation [175].

Under hypoxia, the levels of ROS, NO, and class I non-symbiotic hemoglobin increase in plant cells. NO levels are directly dependent on the plant’s class I non-symbiotic hemoglobin [176]. Hemoglobin and NO facilitate an alternative respiratory pathway for mitochondrial electron transport under oxygen deprivation [177]. In hypoxic conditions, hemoglobin serves as a terminal component for soluble NO dioxygenase activity, increasing nitrate content through the reaction of NO with oxyHB, a process known as the Hb/NO cycle. This cycle helps maintain ATP levels by consuming NADH, although the exact mechanism remains unclear. Overexpression of the Hb gene affects signaling by influencing NO levels, whereas lower Hb gene expression increases ET content, leading to aerenchyma formation. Hemoglobins support alternative respiratory metabolism when oxygen levels are too low for cytochrome oxidase saturation.

Additionally, NO triggers MAP kinase or guanylate cyclase pathways, leading to aerenchyma formation and cell death, which helps plant avoid hypoxia by providing oxygen from the shoots and maintaining energy and redox balance. Hypoxia stress activates hemoglobin through oxygen and nitrite, enhancing NO content and reducing metabolic activity in stressed cells [175]. In anoxic and hypoxic conditions, NO and hemoglobin are interconnected, providing an alternative electron transport chain (ETC) pathway in *O. sativa* [178]. The Hb/NO cycle regulates energy levels and triggers signaling cascades via ET and NO [178].

## 4. Detoxification Mechanisms for ROS in Crop Plants

### 4.1. Overview of Antioxidant Defense Systems in Plants

Antioxidants are molecules that effectively quench free radicals and protect cells. They donate electrons to free radicals and stabilize them or capture the free radicals and inhibit the chain reactions [3,179,180]. Antioxidants play a significant role in plants under stress conditions. They are responsible for modulating free radicals in plants by oxidation-reduction reactions. Antioxidants prevent oxidative bursts and neutralize the existing radicals [4]. Antioxidants are broadly classified into enzymatic and non-enzymatic antioxidants.

### 4.2. Enzymatic Antioxidants and Their Roles

Enzymatic antioxidants include CAT, SOD, POD, GR, GPX, GST, MDHAR, DHAR, and APX [181,182,183]. The production and the activity of enzymatic antioxidants increase on exposure of stress. But the decrease in the activity of antioxidant enzymes under heavy metal toxicity was reported in crops such as *Sorghum bicolor* (L.) Moench, *Zea mays* L., *A. thaliana*, *Cicer arietinum*, *Gossypium hirsutum*, and *Biscutella auriculata* [184,185,186]. This depends on the metal concentration, duration of incubation, and structure of enzymes [186]. Excessive ROS-mediated oxidation of biomolecules and structural damage and inhibition of enzymes are the major reasons for reduction in enzyme activity.

#### 4.2.1. Superoxide Dismutase (SOD)

The activity of SOD can be considered as the first step taken by the plants to tolerate abiotic stress. It belongs to the family of metalloenzymes. The genes coding for SOD are rapidly expressed under abiotic stress conditions and enhancing its production. SODs have been classified into three groups based on their associated metal cofactors: Cu/Zn SOD; Fe/SOD; and Mn-SOD. The most abundant enzyme is Cu/Zn-SOD. These enzymes are localized in the subcellular compartments including chloroplasts, mitochondria, peroxisomes, and cytosol, which are the main sites of ROS production. The structure of SOD consists of two domains. The secondary structure has β-sheets and α-helices. The α-helices dominate in Fe and Mn-SOD, while Cu/Zn-SOD is dominated by β-strands [180]. They dismutate superoxide radicals as shown below

$$O_{2}^{\cdot -}+O_{2}^{\cdot -}+{2H}^{+}\to {2H}_{2}O_{2}+O_{2}$$

The oxidation-reduction reaction results in the production of oxygen and H_2_O_2_ [33]. Zameer et al. [187] showed that *Daucus carota* has nine SOD genes consisting of five *Cu/ZnSOD*s, two *FeSOD*s, and two *MnSOD*s that distributed across five chromosomes. These enzymes have a similar protein and gene structure. The cis-regulatory element present in the promoter region may control their differential expression. Huo et al. [183] reported that *N. tabacum* has 15 SOD genes, including *7FSDs*, *5CSD*, and *3MSDs*. Plant hormones, TFs, and stress-related signals control their expression. Plants tolerant to environmental stress have higher expression levels of SOD genes.

#### 4.2.2. Catalase

The enzyme CAT plays a significant function in plant defense, aging, and plant development. It controls the free radicals generated through photorespiration, fatty acid oxidation, and mitochondrial electron transfer [188,189,190]. It efficiently dismutates H_2_O_2_ into water and oxygen. This enzyme has been found in peroxisomes, which are the main site for H_2_O_2_ production. It is also localized in mitochondria, chloroplasts, and the cytosol. The production of CAT in plants is directly related to factors like duration, time, and intensity of stress [25].

$$2H_{2}O_{2}\overset{Catalase}{\to }2H_{2}O+O_{2}$$

Catalases have been categorized into three groups: CAT I, CAT II, and CAT III. The first one is mostly localized in photosynthetic tissues and controls the H_2_O_2_. The second one exists in vascular bundles and is involved in lignification, while the third-class CAT removes the radicals produced by fatty acid degradation in the glyoximes [191]. CAT exists as dimers and tetramers in which a small gene family encodes the monomers. The monomers may be identical or different in different plants. CAT exists in different isoforms, and their properties may vary even in the same plant [192].

#### 4.2.3. Peroxidase

Peroxidase is present in the cytosol, and vacuoles and is synthesized by the Golgi apparatus and endoplasmic reticulum. X-ray crystallography techniques have been used to explore POD structure in *Hordeum vulgare*, *Glycine max*, *Arachis hypogea*, *A. thaliana*, and *Armoracia rusticana*. The POD structure contains one polypeptide chain with 300 amino acids, two calcium atoms, and iron (III) protoporphyrin IX. It also contains disulfide linkages and salt-bridge motifs. The carbohydrate content (N-linked glycan) in the structure can be more than 25% [193]. There are three cycles in which ROS is removed and generated in the cells, i.e., peroxidative, oxidative, and hydrolytic cycles. During these cycles, new ROS are generated and eliminated. The free radicals interact with plant cell walls and retard plant growth to conserve the available resources in stress conditions [194].

$${Fe}^{3+}\overset{H_{2}O_{2}\to H_{2}O}{\to }{Fe}^{4+}=O\overset{{AH}_{2}\to \cdot AH}{\to }{Fe}^{4+}-OH\overset{\cdot AH\to \cdot A+H_{2}O}{\to }{Fe}^{3+}$$

Peroxidase converts H_2_O_2_ into water by the oxidation of ferric ions. In the second step, phenolic compounds oxidized the Fe^4+^O and made Fe^4+^OH. In the last step, the enzyme is regenerated. These reactions are carried out by multiple types of PODs. The types of PODs found in plants include guaiacol peroxidases, APXs, PRXs, and GPXs [195].

### 4.3. Non-Enzymatic Antioxidants and Their Roles

The non-enzymatic antioxidants include phenolic compounds, flavonoids, carotenoids, AsA, GSH, tocopherols, polysaccharides, polyamines, minerals, vitamins, and amino acids [196].

#### 4.3.1. Ascorbic Acid

Ascorbate is a low-molecular-weight antioxidant found in the cytosol, chloroplasts, mitochondria, peroxisomes, endoplasmic reticulum, and vacuoles. It plays an essential role in mitosis and the production of ethylene (ET) and zeaxanthin. It effectively reduces H_2_O_2_ and ^1^O_2_ [197]. They also scavenge ROS through the glutathione–ascorbate cycle, which involves enzymes such as GR, MDHAR, APX, and GR. It runs at four locations, including chloroplasts, peroxisomes, mitochondria, and cytosol. Ascorbate directly reacts with H_2_O_2_ and oxygen-free radicals and protects the cell organelles [198]. APX reduces H_2_O_2_ into water and protects the membranes. It also reacts with the tocoperoxyl radical and regenerates vitamin E [199].

#### 4.3.2. Glutathione

GSH is a small but strong tripeptide antioxidant found in mitochondria, chloroplasts, endoplasmic reticulum, vacuoles, and cytosol. It scavenges the H_2_O_2_, ^1^O_2_, oxygen-free radicals, and hydroxy radicals through the ascorbate–GSH cycle [60]. Chloroplasts have the highest concentration of GSH. It is involved in the synthesis of phytochelatins, which stabilize heavy metal ions and reduce ROS production in plant cells [200]. It maintains the reduced state of zeaxanthin and α-tocopherol, prevents protein denaturation, and acts as a substrate for GPX and GST (both involved in ROS detoxification).

#### 4.3.3. Carotenoids

Carotenoids are the lipid-soluble accessory pigments that act as antioxidants. They are localized in plastids. They have been categorized into two main groups, xanthophylls and carotenes. Xanthophylls are more polar due to the presence of oxygen atoms, which include zeaxanthin, neoxanthin, violaxanthin, fucoxanthin, and lutein. Carotenes include lycopene and β-carotene, which are more prominent than other compounds. Carotenoids quench free oxygen radicals in chloroplasts (generated during photosynthesis) by physical and chemical mechanisms [196]. These pigments chemically react with free radicals and delocalize the unpaired electron. Moreover, they react with the product of lipid peroxidation and terminate the chain reaction. They respond to oxygen-free radicals and dissipate excess excitation energy as heat. Carotenoids are electron-rich structures, and they absorb the excess energy of ^1^O_2_ and release that energy as heat [60].

#### 4.3.4. Phenolic Compounds

Phenols show higher reactivity with free radicals as compared to ascorbate and tocopherols. The mechanism of action includes the donation of electrons, delocalization or stabilization of unpaired electrons, and inhibition of the Fenton reaction. They act as chelators for redox-active metal ions and non-redox-active ones, which are responsible for terminating free radical chain reactions. They scavenge H_2_O_2_ and other free radicals. They rapidly donate electrons at the site of lipid peroxidation, and their intermediate phenoxy radicals are relatively stable and even react with other free radicals to terminate their chain reactions [191].

The low-molecular-weight phenolics were called flavonoids and categorized into subclasses like flavanones, flavones, isoflavones, anthocyanins, and flavanols. They quench ROS, including OH^•^, O_2_^•−^ anions, ^1^O_2_, and peroxyl radicals. They donate hydrogen ions or electrons and chelate free metal ions by forming stable complexes. They also suppress the activity of enzymes functional in the biosynthesis of free radicals [2,201,202].

### 4.4. Synergistic Linkage of the Components of the Antioxidant Defense System

Different antioxidants work together in a synergistic way to improve plant responses against stress conditions. Their interaction depends on structure, concentration, and ratios. Antioxidants can form stable complexes with higher antioxidant potential than their precursors. Moreover, they promote the synthesis of new phenolic compounds or dimers and regenerate stronger antioxidants [203].

**Figure 2:**
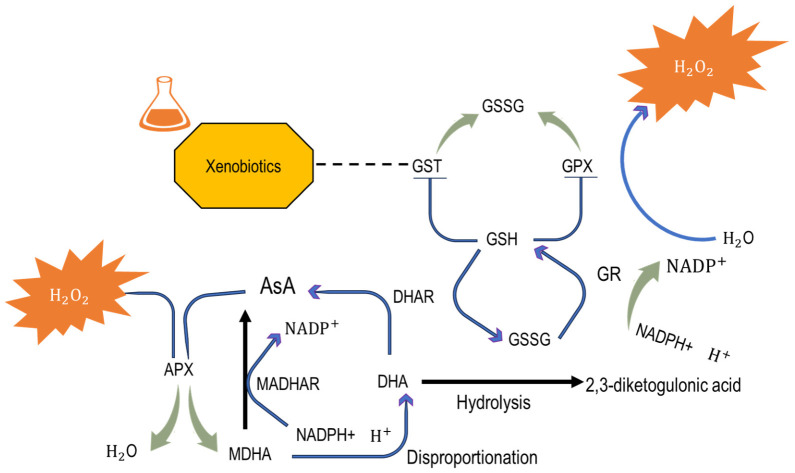
Illustration of the ascorbate–glutathione cycle.

Different enzymes work together in the AsA-GSH cycle to detoxify H_2_O_2_ (Figure 2)_._ APX uses AsA as an electron donor and reduces H_2_O_2_ into H_2_O. The AsA was oxidized and became monodehydroascorbate (MDHA). Then MDHAR converts MDHA into AsA by using NADPH as a source of energy, and MDHA is also disproportionated into dehydroascorbate (DHA). The DHAR converts DHA to AsA by using GSH, or it is hydrolyzed into 2, 3-diketogulonic acid. The enzyme GR converts GSH to glutathione disulfide (GSSH) by using NADPH.

This cycle transfers electrons from NADPH to H_2_O_2_. GPX uses GSH to reduce H_2_O_2_ into H_2_O and oxidizes GSH to GSSG. GST also catalyzes xenobiotics by using GSH [204,205].

SOD and CAT work together to detoxify O_2_^•−^ anions. SOD converts O_2_^•−^ into H_2_O_2,_ and CAT breaks H_2_O_2_ into water and oxygen.

$$O_{2}^{\cdot -}\begin{array}{c} 2H^{+},e\\ \to \\ SOD \end{array}H_{2}O_{2}\begin{array}{c} \left(2\right)\\ \to \\ CAT \end{array}2H_{2}O+O_{2}$$

**Figure 3:**
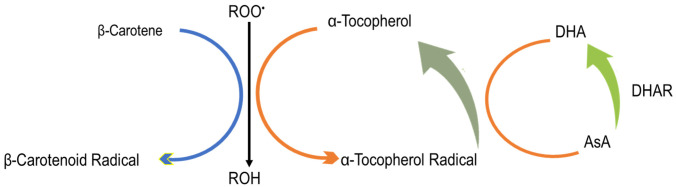
Role of non-enzymatic biomolecules in ROS scavenging.

The non-enzymatic antioxidants are also supports to detoxify the free radicals produced by lipid peroxidation in plants (Figure 3). The antioxidants β-carotene and α-tocopherol become oxidized and transfer the electrons to neutralize free radicals. The β-carotene radical and α-tocopherol radical are relatively stable. The α-tocopherol radical forms an intermediate by reacting with other unstable free radicals. AsA regenerates α-tocopherol from its radicle form [98].

The antioxidant component in green tea has high ROS scavenging activities. Farooq and Sehgal [206] reported a strong interaction between the non-enzymatic antioxidation, particularly the phenolic content of *Ocimum gratissmum* and green tea. The different ratio of their binary mixture shows a correlation with antioxidant enzyme activities. Kaempferol and Epigallocatechin gallate (EGCG) are present in many plants, and they are rich sources of flavonoids. Their antioxidant activities impart beneficial effects on human health. The combination of kaempferol and EGCG improved the antioxidant activities of POD, SOD, and GPX that suppress the ROS efficiently [207].

## 5. ROS Signaling and Molecular Cross-Talk in Response to Abiotic Stress

### 5.1. ROS as Signaling Molecules

The detrimental effects of ROS molecules have been widely documented, but the positive roles of these signaling molecules require further exploration. The impact of ROS molecules on cell growth regulation, the cell cycle, apoptosis, signaling of phytohormones, tolerance mechanisms of plants under biotic and abiotic stresses, and developmental processes has been evaluated in several studies [208].

**Figure 4:**
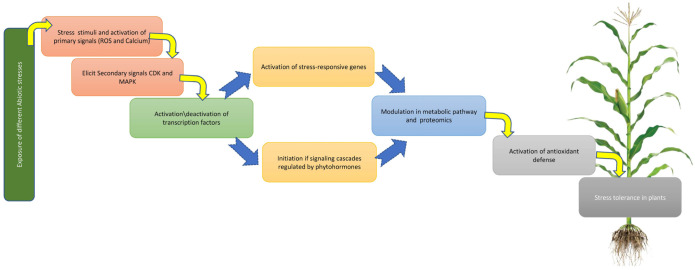
ROS-induced cellular events in abiotic stress tolerance.

These highly reactive molecules participate in the signaling nexus with Ca^2+^ and plant hormones in regulating redox homeostasis events, which finally determine the response of plants towards their environment [209]. This interconnection is represented in Figure 4.

The impact of ROS molecules as signaling factors has been explained using the term “redox biology”, where these molecules aid in regulating and maintaining fundamental cellular processes in plants [4]. The detailed view on the molecular mechanisms operational in the regulation of antioxidant genes; the interactions of signaling molecules such as MAP kinases, PI3 kinase, PTEN, and protein tyrosine phosphatases; mitochondrial oxidative stress; programmed cell death; aging; iron–sulfur cluster proteins (IRE–IRP) mediated iron homeostasis; and DNA damage regulated by *ATM* (ataxia telangiectasia mutated) are essential to modify a crop performance towards different stresses. Impacts of different stresses and ROS accumulation on different crop plants are summarized in Table 1.

Moreover, it is crucial to summarize the interaction between different signaling molecules, such as NO, H_2_O_2_, and other ROS, H_2_S, and Ca^2+^, to predict transcript-level expression of specific genes and proteins [220]. Similarly, ROS molecules induced the activities of auxin, ABA, SA, ET, SOD, CAT, APX, MDHAR, GPX, and melatonin. Nevertheless, this signaling network affects the expression and antioxidant activity under stress conditions [221].

Recent studies have illuminated the intricate spatial and temporal regulation of ROS in crop plants under sequential and combined abiotic stresses, revealing how plants finely tune ROS signaling to distinguish between acclimatory responses and oxidative injury [26,220,221,222,223,224,225]. Under sequential stresses, such as drought followed by salinity, ROS production is initially localized to specific cellular compartments like chloroplasts and peroxisomes, thereby initiating localized signaling cascades that activate stress-responsive genes. However, if these stresses persist or overlap, ROS levels can escalate, leading to widespread oxidative damage. Plants mitigate this by spatially restricting ROS accumulation and enhancing antioxidant defenses in critical areas, thereby preserving cellular integrity and function.

Central to this regulation are redox-sensitive TFs that integrate ROS signals with hormonal pathways, particularly ABA, salicylic acid (SA), and jasmonic acid (JA). ABA-induced ROS accumulation maintains equilibrium in ROS generation as well as the antioxidant activity in a specific pattern [226]. These TFs, such as MYB and ERF families, undergo oxidative modifications that alter their DNA-binding activity and interaction with cofactors, thereby regulating the expression of target genes involved in stress acclimation. Additionally, hormonal cross-talk further refines this regulation; ABA often promotes stomatal closure and osmotic adjustment, SA enhances disease resistance, and JA coordinates defense responses against herbivory. Together, these mechanisms enable plants to cop up with non-optimal environmental conditions by balancing ROS signaling to promote acclimation while preventing oxidative damage [227,228,229].

### 5.2. ROS and Hormonal Signaling Cross-Talk

The signal integration between ROS molecules and different hormones (auxin, cytokinins, ET, ABA, and gibberellins (GA)) aids plants withstand abiotic stress conditions [230].

Hormones and stress response signaling nexus are significantly involved in the generation, detoxification, and transport of ROS molecules (Figure 4). An integrated regulation of phytohormone levels with ROS affects different developmental processes in plants [231]. A complex signaling network between ROS molecules and phytohormones influenced root development, and the modified root architecture aids in increasing the tolerance level of plants [232]. Like the phytohormones, other plant growth regulators such as brassinosteroids (BRs), strigolactones, SA, NO, melatonin, polyamines, and hydrogen sulfide have a crucial role in the ROS signaling mechanisms [233].

Abiotic stress-induced ET accumulation and the impact of this gaseous phytohormone on the physiological, metabolic, molecular, and anatomic adaptations of plants with ROS regulation and interactions with other phytohormones were scrutinized in different studies [234]. Of the different abiotic stresses, waterlogging stress induced ET-induced modulations in plants, and the novel insights on ET-induced gene activation/regulation help to manipulate the plant genome to create desirable plants [234]. Petal abscission in rose plants is dependent on ET production and ROS accumulation. A cross-talk between ROS and ET through the differential expression of specific genes was evaluated in rose petals [235].

Major genes involved in the signaling cascades related to ROS production are *RhRHS17*, *RhIDH1*, *RhIDH-III*, *RhERS*, *RhPBL32*, *RhFRS5*, *RhRAC5*, *RhRBOHD*, *RhRBOHC*, and *RhPLATZ9*. The repression of *RhCCR4*, *RhUBC30*, *RhSOD1*, *RhAPX6.1*, and *RhCATA* genes was also involved [235]. *O. sativa* forms lysigenous aerenchyma in roots to cope with hypoxic conditions as it lives in a water-logged environment. These hypoxic conditions promote ET accumulation that stimulates aerenchyma formation through programmed cell death dependent on NOX or respiratory burst oxidase homolog (RBOH) H-mediated ROS molecules [236].

ROS treatment could activate ET synthesis and the ET signaling pathway through a chain of cellular events [237]. Degradation of damaged mitochondria is the major event among them, as it is the major organelle producing ROS, and it supports the survival of *A. thaliana* at waterlogging stress. The induction of ET-related genes (*ACO2*, *ACS2*, *ERF72*, *ERF73*, and *EIN3*) and an increase in ROS accumulation were observed as parallel events in *A. thaliana* roots, and these together help to activate the antioxidant defense system [238].

ABA is another phytohormone actively participating in the regulation of downstream stress machinery by triggering various processes. ABA is considered the premier signal for stress, and it modulates transcriptional and post-transcriptional genes, as well as physiological and metabolic processes [239]. In ABA-dependent stress response processes, TFs such as *DREB2A/2B*, *AREB1*, *RD22BP1*, and *MYC/MYB* are precisely active through interacting with their corresponding cis-acting elements such as *DRE/CRT*, *ABRE*, and *MYCRS/MYBRS*, respectively.

The apoplast ROS production is modulated by ABA, and these highly reactive molecules are involved in the regulation of the ABA signaling pathway [240]. ABA-mediated stress tolerance mainly initiates with the closure of stomata, and it was shown that ABA induces H_2_O_2_ biosynthesis in guard cells of stomata by the involvement of NOX, leading to the closure of stoma by regulating Ca^2+^ channels in the cell membrane [239]. The stomatal closure depends on a complex signaling network between ABA, ROS, and Ca^2+^.

Under drought, water deficit signals were perceived by roots, and induced the production of CLAVATA3/EMBRYO-SURROUNDING REGION RELATED 25 (CLE25) peptides. This peptide is moved to the guard cells to augment biosynthesis of ABA. Perception of ABA signals aids in inactivating PP2C, which stimulates the activation of the protein kinases SnRK2s. ABA-activated SnRK2s increase apoplastic ROS generation outside of guard cells and transport it into the guard cells. The receptor kinase, HYDROGEN PEROXIDE-INDUCED CA^2+^ INCREASES1 (HPCA1), is induced by apoplastic ROS, which activate Ca^2+^ channels in the guard cell membranes [241].

Of the different ROS receptors, the receptor of H_2_O_2_ plays a crucial role in stomatal closure [212]. Moreover, ABA could enhance ROS scavenging by activating antioxidant enzymes of plants such as SOD, POD, CAT, APX, and GR. The antioxidant cascades regulated by ABA are an important stress tolerance mechanism operational in *O. sativa* plants [242].

Furthermore, a cross-talk between ABA and ROS molecules can induce gene expression, biosynthesis of compatible solutes, hypersensitive actions, and programmed cell death in a stress-exposed cell [243]. Increased ROS scavenging, reduced membrane leakage, and vacuolar compartmentalization of toxic radicals are other cellular events stimulated by the ABA-ROS nexus [244]. Under cadmium stress, a complex network between ABA, auxin, BRs, and ET, and signaling molecules like NO, H_2_O_2_, and hydrogen sulfide was observed to balance the antioxidant system of plants [245].

Auxin is another phytohormone related to the ROS signaling pathways. ET-dependent aerenchyma formation is connected to AUX/IAA-mediated auxin signaling as well as ROS signaling cascades. Current studies have proved that the RBOH-ROS-auxin signaling cascade aids in modifying root development during exposure to various stresses [237].

With root morphology, ROS is an essential factor determining intercellular flow and distribution of the phytohormone auxin. In *A. thaliana* roots, the synchronized application of auxin as well as ROS increased lateral root formation. Exogenous ROS treatment modulates the endogenous auxin level and rate of cell division [238]. Studies in *O. sativa* roots helped to understand auxin-ROS cross-talk during different developmental stages of roots, and it also explained the activation of antioxidant genes from POD, GR, GST, and TRX reductase families.

Comparison of global database, laser capture microdissection-RNA sequencing, and RNA in situ hybridization simultaneously proved the role of auxin-ROS cross-talk in root organogenesis and stress tolerance [246]. IAA application in salt-stressed seedlings increased ROS detoxification and activities of the antioxidant enzymes, and prevented lipid peroxidation [247]. Of the auxin signaling components, auxin efflux transporters also play important roles in drought and salt tolerance and, hence, may be exploited to breed drought-tolerant crops. Also, ROS, along with peptide hormone and auxin signaling, are essential in root growth under stress [248].

Another phytohormone, cytokinin, is also involved in the mitigation of abiotic stress intensity in plants by regulating cytokinin-targeted gene expression [249]. The rate of transcription of these genes was dependent on a series of phosphorylation cascades that pass a signal to specific TFs [250]. Cytokinin-induced stress tolerance is dependent on the interaction of histidine kinases and more axillary growth 2 (MAX2) by affecting ABA response, ROS homeostasis, and leaf hydration [251].

Moreover, a cross-talk between cytokinin and IAA enhanced catalytic functions of enzymes that participate in the AsA-GSH cycle (APX, GR, DHAR, and MDHAR) and that help to mitigate oxidative stresses associated with complex environmental conditions [252]. Similarly, the cross-talk with cytokinin (6-benzylaminopurine) and methyl jasmonate also interacted with ROS signaling mechanisms and antioxidant defense [253]. The cross-talk between cytokinin and other phytohormones, including ABA and ET, was investigated, and it supported the plant to survive under salt stress [254]. Application of CPPU (*N*-(2-chloro-4-pyridyl)-N′-phenylurea), a phenylurea cytokinin, on *O. sativa* plants aids in knowing the differential and selective expression of some TFs during drought stress [255].

Gibberellic acid, a tetracyclic diterpenoid phytohormone, regulates many growth and developmental stages of plants. Exogenous and endogenous levels of GA also serve as stress defenders in plants. The effects of the exogenous GA application on plants (Sorghum seedling) were prominent in the modulation of morphological and physiological responses by the regulation of metabolic pathways and salt tolerance regulatory genes [256]. Moreover, signaling pathways in GA and GA-mediated cross-talk with other plant hormones are important aspects that promote the antioxidant defense of crops [257].

Analysis of the impact of GA on ROS homeostasis indicated that the GA regulated the metabolism of ROS and decreased the generation of O_2_^•−^, H_2_O_2_ content, and MDA content. Moreover, it improved the catalytic potential of SOD, POD, and CAT, with AsA and GSH content under drought stress [258]. Similarly, the use of GA significantly enhanced the expression levels of APX, GR, and Fe-SOD under weak light stress [259].

**Figure 5:**
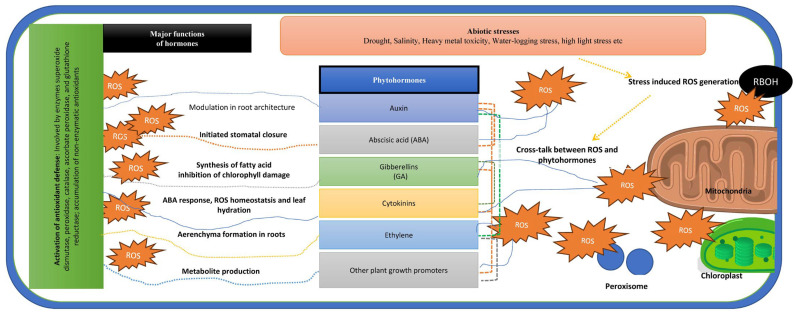
Interactions of ROS molecules and phytohormones in abiotic stress tolerance (Dashed lines indicates inter-connections).

Under antibiotic, ciprofloxacin (CIP) toxicity, minor GA content aided in reducing the oxidative stress. Higher GA content induced the expression of TFs that trigger the synthesis of eicosanoids, isoprenoids, and fatty acids, which are antioxidants in function [260]. It was noticed that the Cd stress (in *Nicotiana tabacum*) altered the levels of GA and BR levels with an inhibition of chlorophyll breakdown and reduction in ROS levels [261]. Application of other plant growth regulators during abiotic stress aids the plant to cop up with it by modulating physiological and molecular processes [253]. Interactions of ROS molecules with different phytohormones are represented in Figure 5.

### 5.3. ROS and Calcium Signaling

Calcium is an essential secondary messenger that coordinates multiple cellular processes and enhances the stress tolerance of plants [262]. Accumulation of osmoprotectants, antioxidant boosting, regulation of polyamines and NO machinery, and pH regulation are the important tolerance mechanisms stimulated by Ca^2+^-mediated signaling [263].

**Figure 6:**
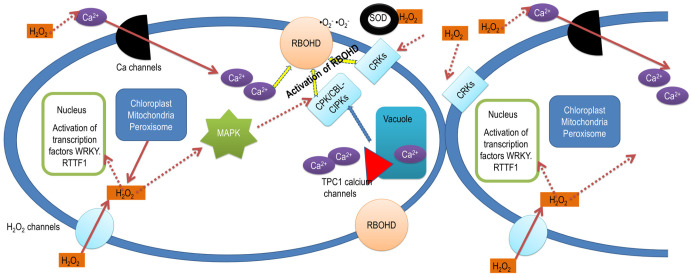
Cross-talk between ROS, Ca^2+^, and RBOH. Accumulation of ROS (H_2_O_2_) induces Ca^2+^ channels which increase cellular Ca^2+^ levels, and the RBOH is activated by Ca^2+^-dependent protein kinases (CPK/CBL-CIPKs, Cys-rich receptor kinases (CRKs), or directly by Ca^2+^. RBOH is involved in the generation of superoxide anion (O_2_^•−^), which transforms to H_2_O_2_ by the action of SOD. Further, this H_2_O_2_ induces Ca^2+^ channels of neighboring cells.

Modulation in Ca^2+^ level during stress is referred to as “signature”, which indicates the stimulus-induced signal changes able to detect, decode, and transmit to elicit different cellular responses [264]. Because of environmental stress, Ca^2+^ signatures will be formed in cells, and these signatures are perceived by Ca^2+^ sensors. Ca^2+^ sensors and relay proteins are involved in downstream signaling mechanisms to decode the Ca^2+^ signatures [265]. Sensor-relays (Calmodulin (CaM), CMLs, calcineurin B-like proteins (CBLs)) and sensor-responders (Ca^2+^-dependent protein kinases (CPKs/CDPKs)) are Ca^2+^ sensing elements critically regulating the ROS production in response to stress (Figure 6).

Variation in the concentration of Ca^2+^ in vacuoles, endoplasmic reticulum, mitochondria, chloroplasts, nuclei, and the cell wall aids cellular communication [266]. The Ca^2+^ level in the cytoplasm is maintained by Ca^2+^ transporters such as Ca^2+^ATPases, P1-ATPases (HMA1), the mitochondrial Ca^2+^ uniporter complex (MCUC), and Ca^2+^-exchangers (CAXs). Among them, Ca^2+^-ATPases are involved in developmental processes, signaling mechanisms of hormones, stomatal opening, fertilization, and stress signaling in plants [267].

An interaction between Ca^2+^ level and ROS content synergistically coordinates the signaling cascades for different cellular events, which supports different stress tolerance responses (Figure 6). Cell-to-cell communication is established with the direct interaction of ROS and Ca^2+^ signaling pathways, facilitating long-distance transmission of signals in cells [268]. This indicates that Ca^2+^-ROS cross-talk helps to transmit information regarding the redox state of one cell to all other cells of an organism.

Ca^2+^ induced ROS production (CIRP) and ROS-induced Ca^2+^release (RICR) are two critical steps that help to cope with stress [269]. Exposure to abiotic stress leads to the enhancement of ROS that induces oxidative stress, which activates the Ca^2+^ pathway. This Ca^2+^ signaling induces the apoplastic ROS production that further enhances the Ca^2+^ level of the cell through a vacuolar Ca^2+^ release system composed of TPC1. The feed-forward loop of RBOH is then stimulated by further cycles of Ca^2+^ influx and propagation to the closest cells.

Activation of RBOH protein significantly depends on the level of Ca^2+^ by the binding of Ca^2+^ to EF hand motifs, as well as Ca^2+^-dependent phosphorylation. RBOH activity could be promoted by the influence of Ca^2+^-dependent protein kinases (CPK/CBL-CIPKs and Cys-rich receptor kinases (CRKs). ROS generated during stress activates a large spectrum of ion channels involved in stress tolerance, and these channels support cycles of Ca^2+^-triggered ROS generation that are interconnected to ROS-dependent Ca^2+^ fluxes [266]. Moreover, this signaling cascade leads to the stimulation of antioxidant defense for promoting ROS scavenging [269].

### 5.4. ROS and MAPK Signaling Pathways

Stress signaling of plants is achieved through complex cellular events directed by specific proteins. The mitogen-activated protein kinase (MAPK) is the fundamental protein among them, which is involved in plant growth, development, yield, abiotic and biotic stress adaptation [270]. In a cascade of cellular events that eventually enhance the tolerance of plants, the receptors of the cell surface get the stimuli and transfer these stress signals to various pathways by the regulation in phosphorylation processes, which are controlled by MAPK and secondary messengers [271]. Regulation of gene expression, cell activities, and protein functions is the major role of MAPK, leading to specific stress-mitigating responses. MAPKs have crucial roles in governing different inevitable cellular functions such as proliferation, differentiation, stress adaptation, and apoptosis [220]. MAPK, with phosphatases, acts as the key factor regulating cytoskeletal proteins in the cytosol or TFs in the nucleus [270].

MAPK kinase kinases (MAPKKKs), MAPK kinases (MAPKKs), and MAPKs are the major elements of signaling cascades that improve stress tolerance by transcriptional reprogramming [272]. The signaling event starts with the activation of a MAPKKK induced by ROS accumulation, which activates an MAPKK. It further activates an MAPK. The importance of conserved domains has to be explained in these specific linear events of activation processes.

**Figure 7:**
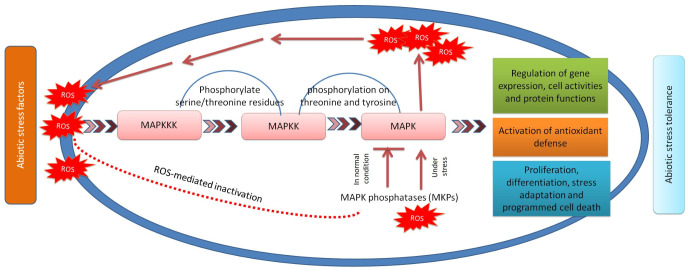
ROS involved regulation of the MAPK signaling pathway directly by activating upstream kinases such as MAPK kinase kinases (MAPKKKs), MAPK kinases (MAPKKs), and MAPKs and indirectly by inhibiting MAPK phosphatases.

The MAPKKK activated by ROS stimuli phosphorylates some conserved amino acid residues within MAPKK. The consensus motif is with two serine/threonine residues (S/TxxxxxS/T). Phosphorylated MAPKK leads to the activation of MAPK by phosphorylation on threonine and tyrosine residues in the T(E/D)Y conserved motif. Different from this pathway, in an indirect pathway, the ROS is able to modulate MAPK signaling [273]. In this pathway, the long-term activation/phosphorylation of MAPK was achieved by the inactivation of MAPK phosphatases (MKPs). Moreover, in a cyclic episode, MAPKs themselves can generate ROS as part of their signaling by the expression of ROS-producing enzymes (Figure 7).

MAPK signaling cross-talk with ROS and ABA under abiotic stress induces adaptability in plants. Signal molecules like phytohormones and ROS activate the MAPK cascade, the activation of the antioxidant system, and synergistic cross-talk between different signaling molecules to regulate plant responses to abiotic stress [273]. Fascinatingly, MAP kinases have a positive/negative role in stress tolerance. Most of the time, they up-regulate stress-mitigating factors, but some MAP kinases have negative roles in stress, and they increase ROS accumulation.

### 5.5. Transcriptional Regulation and Signal Transduction

Tolerance mechanisms of plants towards extreme environmental conditions are crucially dependent on transcriptional regulation/modulation in gene expression. It is the process of controlling gene expression by regulating the transcription of DNA into RNA [274]. The regulation is mainly associated with the enzyme RNA polymerases, and eukaryotes have Pol I, Pol II, and Pol III [275]. Histone remodeling enzymes, TFs, enhancers, and repressors have a crucial function in the catalytic action of RNA polymerases, and they regulate the accessibility of DNA to the enzyme. The impact of TFs is crucial, and it is elaborated here.

Transcription factors (regulons) are proteins that play a significant role in the modulation of gene expression by binding to target gene promoters [276]. Interaction between various signal transduction pathways that are regulated by phytohormones is controlled by the activation/deactivation of TFs. Various TFs in plants participate in the regulation of ROS metabolism. ET response factor (*AP2/ERF*), *WRKY*, *NAC*, and *MYB* are the major families of TFs involved in the signaling cascades that activate the enzymatic antioxidant defense network [277]. Similarly, plants have non-enzymatic defense mechanisms, which include metabolites such as tocopherol, flavonoids, proline, and carotenoids. This non-enzymatic pathway is controlled by TFs such as *bHLH*, *MYB*, *bZIP*, and *Dof*.

The family of *WRKY* TFs is involved in the regulation of stress-related gene (*RD29A*, *HSP90* (heat shock protein 90), *DREB2A*, *DREB2B*, *CuZnSOD*, *NCED1* (nine cis epoxycarotenoid dioxygenase 1), *NCED3*, *NCED6*, *LEA5*, *NtP5CS*) expression, ROS-related gene (*RbohD*, *CSD1*, *CSD2*, *FSD3*) expression, and hormone signaling gene (*EIN3*, *ABF3*, *ABF4*) expression. This gene’s action helps to activate the ROS scavenging system, the catalytic activity of antioxidant enzymes (POD, SOD, CAT), and lipid peroxidation rate under different abiotic stresses such as cold, drought, and salinity [278].

NAC TFs regulate the accumulation of proline and glycine betaine, carotenoid biosynthesis, and activities of antioxidant enzymes to cope with abiotic stresses. Induction of stress marker genes and ROS scavenging enzyme genes is regulated by the ERF74-RbohD-ROS signal pathway, and the AP2/ERF TFs family is the major controller of this pathway [279]. Moreover, ERF6 functions as a transcriptional activator and suppressor of genes in response to abiotic stress, and it significantly increases CAT and GPX activities with the GSH and proline content.

Another important TF family, MYB, is also involved in the mitigation of oxidative stress. *CmMYB012* (*Chrysanthemum morifolium*), *SlMYB49* (tomato), PlMYB108 (*N. tabacum*), and *TaMYB86B* (*Triticum aestivum*) are identified as crucial factors for stress tolerance by improving flavonoid content and antioxidant enzyme activity [277]. Induction of the *NtbHLH123*-*NtRbohE* signaling pathway, up-regulation of the antioxidant genes SOD and POD, reduced proline biosynthesis, decreased ROS accumulation, and induced enzyme catalytic potential are the major functions of the TF family *bHLH* [264].

A study was conducted on *O. sativa* plants to analyze the impact of cytokinin on drought stress tolerance, and the results indicated that a large number of TFs were induced by the application of cytokinin. *ONAC*, *MYB*, *bZIP*, *ZFP*, *HD-XIP*, *HOX*, *ROC*, *HSP*, *HSF*, *TIFY*, *WRKY*, and *AP2/ERF* are the major TF families stimulated in *O. sativa*. In contrast, the expression of many drought/ABA-induced TFs like *ONAC002*, *ONAC010-2*, *ONAC053*, *ONAC074*, *ONAC076*, *MYBAS2*, *MYB30*, *GAMYB*, *bZIP2*, *bZIP10/ABI5*, *HD-ZIP HOX8*, *HD-ZIP HOX9*, *HD-ZIP TF1*, *ZFPs* (*CCCH13*, *CCCH16*, *CCCH23*, *CCCH32*, *CCCH34*, *CCCH44*, *CCCH45*, *CCCH51*, *CCCH53*, *CCCH55*, *CCCH58*, and *CCCH67*), *TIFY2b*, *WRKY24*, *WRKY71*, and *bHLH94* were reduced in *O. sativa* due to cytokinin application [256].

The regulatory nexus of ROS metabolism with the involvement of the TFs has a vital role in the balancing of oxidative stress. Exploration of the mechanism operational between TFs and ROS helps to develop novel strategies to impart horizontal stress survival skills in plants.

### 5.6. Interaction with Other Molecules

Liu and et al. [277] summarized the impact of miscellaneous compounds such as sodium nitroprusside (SNP), NO, phosphorus, gamma-aminobutyric acid (GABA), proline, L-carnitine, p-coumaric acid, and GSH, sucrose, trehalose, and chitosan on the ROS signaling pathway [277].

The interaction of NO with different ROS molecules is considered an essential step in the stress tolerance mechanisms. Initially, NO was regarded as a phototoxic compound, but later, several studies proved the signaling potential of these molecules [280]. Improving seed germination, stabilizing ionic balance, protecting photosynthetic machineries, and boosting antioxidative defense mechanisms are other important roles of NO [281]. External application of NO significantly improved stress tolerance in plants [282]. This molecule regulates cellular processes by interacting with O_2_^•−^, H_2_O_2_ and hydrogen sulfide (H_2_S) [283]. The generation and detoxification of ROS and NO influence each other, where ROS are string inducers of NO biosynthesis [284].

ROS and NO signaling pathways aid the plant to modulate primary metabolism and hormonal cross-talk by regulating gene expression. The reaction between NO and O_2_^●−^ is a crucial one as it causes the generation of peroxynitrite (ONOO^−^). This reaction reduces the bioavailability of both O_2_^•−^ and NO, which will affect ROS-dependent signaling and NO signaling. Peroxynitrite affects protein organization by modifying post-translational events such as the tyrosine nitration, metal nitrosylation, sulfenylation, and *S*-nitrosylation [285]. These protein modifications are considered an important signaling process.

Moreover, *S*-nitrosylation mediated by NO has a significant role in maintaining ROS homeostasis. S-nitrosylation at Cys890 of RBOHD inactivates this enzyme, which is actively involved in the generation of ROS and cellular communication. NO causes tyrosine nitration in SOD, and hence it modifies ROS signaling cascades [283].

Nitric oxide maintains a strong cross-talk with other molecules that modulate crucial physiological processes. Ca-calmodulin, MAPK, ABA, and H_2_O_2_ signaling modulate the antioxidant defense of plants [286].

SNP donates exogenous NO, and this NO reacts with GSH to form S-nitrosoglutathione (GSNO) for regulating ROS scavenging and metabolism. Application of exogenous phosphorus regulated ROS generation from chloroplasts, and it reduced lipid peroxidation by increasing the catalytic efficiency of antioxidant enzymes activities [277]. Similarly, exogenous application of proline, L-carnitine, p-coumaric acid, GSH, sugar (sucrose, trehalose, and chitosan) also alleviated oxidative stress by ROS scavenging, stabilizing membrane-associated PODs and NOX [287]. Polyphenol compounds are functionally active in ROS scavenging, and this complex signaling nexus needs *MYBTFs* for the regulation [288]. GABA is a signaling molecule participating in stress responses of plants by reducing the adverse effects of ROS molecules [287].

## 6. Genetic and Molecular Approaches to Manipulate ROS Pathways and Molecular Cross-Talk for Improved Abiotic Stress Tolerance

Modifying ROS pathways genetically seems a promising approach to boost plant resilience against abiotic stresses, such as water scarcity, salinity, and extreme temperature conditions. Indeed, this approach leverages the dual role of ROS as both damaging agents and key components in molecular signalization triggered in the presence of stressful conditions [144].

Most of these strategies rely on establishing the balance between ROS production and scavenging, to enable plants to better withstand environmental constraints, thereby prompting sustainable agriculture along with the improvement of crop productivity and yield [289]. This acknowledges the dual role of ROS, acting both as damaging agents and as key signaling components during stressful events.

Additionally, the exploitation of genetic engineering with the purpose of improving the cross-talk by targeting specific pathways with the support of omics technologies is essential. The implementation of genome editing techniques, mostly CRISPR/Cas 9, represents efficient approaches to dissect plants’ stress response and enhance their resilience to challenging environments [62].

### 6.1. Genetic Engineering for Enhanced Cross-Talk Efficiency

Genetic engineering aims to bolster plant resilience by manipulating the key signaling networks involved in stress transduction and response in plants [289]. The genetic manipulation, especially transcriptional reprogramming of hormone biosynthesis, signaling, and metabolism, represent a promising strategy to increase crop productivity and stress tolerance [290,291].

Several examples of the effectiveness of this approach have already been highlighted in the literature, with the majority of studies focusing on the disruption of phytohormone content or signaling under controlled conditions, at the laboratory scale. Indeed, with the discovery of the dual role of ROS in stress response in plants and the key role of ROS scavenging systems in the alleviation of the harsh effects of abiotic stress, numerous studies investigated the impact of the overexpression of antioxidant system components on plant response/tolerance to environmental constraints [289]. Thus, enhanced tolerance has been achieved through the constitutive expression of antioxidant encoding genes such as *Cu/ZnSOD*, *APX*, *GR*, *GSH*, *DHAR*, and *D-galacturonic acid reductase* in many plant species, including *Solanum tuberosum*, *Festuca arundinacea*, *Nicotiana tabaccum*, *Arachis hypogaea*, *Ipomoea batatas*, and *A. thaliana* [292,293,294,295,296].

Moreover, satisfying results were obtained with the simultaneous expression of ROS scavenging enzymes genes, as reported previously with Shafi et al. [297]. Indeed, it has been demonstrated by these authors that the co-expression of *RaAPX* and *PaSOD* encoding genes in potato enhanced starch accumulation and promoted plant overall growth, even under salt stress conditions. The higher tolerance index of plants was thereby linked with a significant reduction in ROS amounts due to the high APX and SOD enzyme activity recorded in transgenic plants as a result of the genetic manipulation. The enhanced tolerance was mostly linked to the scavenging activity of antioxidant enzymes, which directly interact with ROS and hence reduce excessive ROS amounts within plant cells [298].

Improved tolerance to abiotic constraints can also be attained through the genetic engineering of genes involved in ROS production, scavenging, and signaling, including those encoding MAPKs and protein phosphatases. [62]. Zhang et al. [299] highlighted the importance of GhMKK5 in the enhancement of *Nicotiana benthamiana* resilience to salt and drought stresses. The authors showed through a reverse genetic approach that the overexpression of this gene leads to the up-regulation of ROS-related genes, which ultimately results in a hypersensitive reaction triggered by the accumulation of toxic amounts of H_2_O_2_ [299]. Another MAPK (from *Gossypium*), the GhMKK1, was also found to be implicated in drought tolerance by the regulation of ROS scavenging [300]. The Raf-like MAPKKK gene, DSM1 gene, increased *O. sativa* tolerance to drought, as shown by Ning et al. [301]. Indeed, the dsm1 mutant exhibited high drought sensitivity and oxidative injuries, along with a substantial increase in ROS damage caused by the reduced POX activity. Meanwhile, its overexpression resulted in a higher tolerance to dehydration at the early stages of plant development [301].

### 6.2. Targeting Specific Signaling Pathways

The genetic targeting of the negative regulators of ROS signaling can also trigger the accumulation of ROS, which, as mentioned before, can be effective in the activation of stress-responsive pathways. One of the examples illustrating the efficiency of this approach is the knockout of ABA receptor genes, which enhanced ABA signaling and subsequent ROS levels management during drought stress conditions, thereby yielding high plant tolerance to water scarcity. This interplay suggests that manipulating negative regulators in the ABA signaling pathway could be beneficial in enhancing ROS production and thus increasing plant resilience [240].

For instance, the suppression of ABA receptor-encoding genes enhanced resistance of plants by improving ABA signaling and the management of ROS. This enables plants to better withstand oxidative damage occurring during stress while continuing to grow [239]. Another example is provided with the *A. thaliana* abi1 mutant, lacking functional ABA signaling, which exhibited an effective failure in stomatal closure under drought stress conditions, leading to an excessive water loss along with an enhanced accumulation of ROS [240]. Moreover, it has been demonstrated that ABA signaling interacts with other stress pathways, thereby regulating ROS accumulation. Indeed, as mentioned above, the disturbance of ABA signaling elements can lead to a significant rise in ROS levels. This increase in ROS content plays a crucial role in the stimulation of stress-responsive genes, thus enhancing the plant’s ability to cope with adverse environmental conditions [302].

Other regulatory networks can be explored to enhance plant resilience to abiotic stress through the tight regulation of ROS accumulation. The NOX gene family can be targeted to increase ROS amounts, which can yield a better tolerance to flooding, as previously reported in *O. sativa* by Yamauchi et al. [303]. Indeed, the authors found that the knockout of RBOHH, a member of the NOX gene family, prevented ROS accumulation and induced aerenchyma formation, known for its key role in *O. sativa* adaptation to oxygen-deficient conditions, mostly flooding [303]. Also, enhanced tolerance to drought was assessed in tobacco through the constitutive expression of the wheat E3 ubiquitin ligase *TaPUB1* gene. The transgenic *N. tabacum* plants displayed low ROS amounts, which were correlated with a strong antioxidant activity, thus increasing the survival rate under water deficit conditions [304].

The plant-specific proteins SRO, known for their C-terminal RCD1-SRO-TAF4 (RST), N-terminal WWE domain, and a poly (ADP-ribose) polymerase catalytic (PARP) domain, can actively regulate ROS homeostasis in plant cells. In wheat, the overexpression of TaSRO1 enhanced transgenic plants’ resistance to salinity by regulating ROS homeostasis through ROS-mediated enzymes [62]. In *O. sativa*, OsSRO1 appeared to regulate SNAC1, and enhanced ROS accumulation in plant cells, which can prevent water loss through the active regulation of stomatal closure [305]. Other NAC genes, namely NAC5 and NAC095, were found to be involved in drought and oxidative stress tolerance in *O. sativa*, which can be explored for the improvement of *O. sativa* resistance to these stresses [306].

Overall, these insights suggest that the manipulation of ROS levels through genetic targeting of ROS regulatory networks can significantly enhance plant resilience to abiotic stresses. Future research should be conducted to explore these regulatory networks in order to develop effective strategies for improving crop tolerance and ensuring food security in the face of climate change.

### 6.3. Use of Omics Technologies

The integration of omics technologies, which gather genomics, transcriptomics, proteomics, and metabolomics, has revolutionized the understanding of biological systems and their functioning, particularly in the context of ROS and their involvement in the plant responses towards abiotic stresses. This integrative approach can help to dissect the complex signaling pathways that govern cellular reactions to environmental and physiological stresses [307].

With the emergence of omics technologies, research studies have increasingly focused on the role of genomics in understanding the genetic basis of plant tolerance to stress, with emphasis on ROS production and signaling pathways [307]. In a recent review published in 2024, the authors discussed the importance of genomic tools in the identification of genes associated with ROS production, which are important for developing stress-tolerant crop varieties [308]. Comparative genomics had been used earlier to identify key genes involved in abiotic stress responses in *Cucurbita moschata* [309]. Among the identified genes, *WRKY* and *APETALA2/ET*, responsive factors (*AP2/ERF*) were described as key factors in the regulation of ROS-related pathways, thereby contributing to stress resilience to abiotic stresses. Following the same genomic approach, ROS-specific signaling and detoxification genes were identified in *A. thaliana*, showcasing their critical roles in increasing *A. thaliana* tolerance to drought and temperature fluctuations [310].

Besides genomics, proteomics has been used to decipher the molecular actors involved in ROS-mediated response to abiotic stress in plants. A recent study conducted by [311] underscored the expression of 179 salt-alkali-responsive proteins using qualitative proteome analysis. The identified proteins function in oxidative phosphorylation, the tricarboxylic acid (TCA) cycle, sucrose metabolism, glycolysis, and, more importantly, in ROS homeostasis. Moreover, several antioxidant enzymes, such as CAT and SOD, have been well-characterized using proteomics. Their key role in stress response was identified since they are involved in the maintenance of ROS homeostasis, occurring by the conversion of toxic ROS into less toxic molecules [312]. Studies focusing on redox-sensitive proteins, targeted by ROS during stress responses, showed that these proteins undergo carbonylation during stress exposure. The carbonylated proteins were identified using proteomic techniques, which can be helpful for the elucidation of the molecular mechanisms underlying ROS-mediated signaling in plants [313].

### 6.4. CRISPR/Cas9 and Beyond

CRISPR/Cas9 has emerged as a revolutionary tool to genetically engineer organisms with desired characteristics. Described as the most precise genome editing technique, it allows the introduction of clear-cut modifications at the gene level. This revolutionary tool has been used in agriculture to induce significant and precise modifications of genes involved in different stress signaling pathways, including those associated with ROS signaling pathways and signaling pathway cross-talk [314].

Recent advancements made in CRISPR/Cas9 technology have emphasized its great potential for improving resilience in plants to tolerance to abiotic stresses [315]. While specific examples related to ROS signaling pathway manipulation using this revolutionary genome editing tool are limited, CRISPR/Cas9 technology has been used to modify genes involved in stress responses that often intersect with ROS homeostasis and management. As already highlighted, given the versatile role of ABA in the set of plant responses to environmental constraints, alteration in the ABA signaling components, such as ABA-induced transcription repressors (AITRs) using the highly precise CRISPR/Cas9 system, can impair ROS management, thus affecting stress tolerance [316].

The genome editing of ET-related genes can also be effective in enhancing ROS levels, yielding a better tolerance to abiotic stressors [317]. Targeting the MAPK signaling pathway has also been performed using CRISPR/Cas9 technology, as previously reported by [318]. Indeed, the knockdown of *OsMPK2* enhanced ABA and ROS sensitivity of transgenic *O. sativa* plants, which resulted in a better tolerance to drought stress conditions.

Overall, the above-cited examples highlighted the importance of genome editing tools, mostly CRISPR/Cas9, in enhancing plant resilience to abiotic stresses. They also showed the importance of ROS signaling pathways in the control of ROS homeostasis and content, which can efficiently improve plant resilience against abiotic constraints.

## 7. Harnessing the Benefits of ROS in Plant Stress Tolerance: New Avenue for ROS Research

The dual faces of ROS molecules were discussed in various studies, where one of the faces is the toxic oxidative burst and another one is the signaling nexus. As a positive factor, ROS is an important signaling molecule that regulates different developmental processes and defense mechanisms of plants. When the ROS level exceeds in the cell, it causes damage to the cellular components. ROS molecules in plants under stress and optimal conditions can be measured using chemical probes, biosensors, and electron paramagnetic resonance EPR [319]. These measurements will be used to keep a constant cellular ROS level for stimulating signaling cascades by balancing ROS generation and scavenging.

ROS are generated through different metabolic processes and stress responses, which can orchestrate different signaling pathways by passing the stimuli to TFs, MAPK, phytohormones, and Ca^2+^. Synchronized functioning of ROS-induced metabolic pathways and regulation of gene expressions simultaneously assists a plant to tolerate extreme environmental conditions by activating the antioxidant defense system. The knowledge of the connecting link of different signaling cascades helps to enhance stress tolerance [320]. Regulation of the metabolic pathway by ROS is a crucial step in stress tolerance. Regulation of the primary metabolic pathway depends on the interplay between photosynthetic ROS and stress [321].

Stress-induced leaf senescence can be modulated by the interconnection between sugar metabolism, ROS signaling, and phytohormones, including ABA, cytokinin, 2-Epibrassinolide (EBR), GA, ET, and SA [322]. The influence of ROS on lipid metabolism was also elaborated in microalgae under low-temperature (15 °C) stress [323]. ROS induced the up-regulation of different genes that code the enzymes involved in the different metabolic pathways. Close observation of the interaction between the enzymes, TFs, and ROS gives novel insight into developing new crops with high stress tolerance levels.

Phenols, a key group of secondary metabolites including quercetin, kaempferol, luteolin, apigenin, naringenin, ferulic acid, chlorogenic acid, and caffeic acid, are capable of tolerating stress [288]. ROS stimulates the biosynthesis of phenolic compounds under stress, which involves synchronized enzymatic reactions mainly regulated by phenylalanine ammonia-lyase (PAL) and chalcone synthase (CHS). Moreover, ROS acts as a substrate of lignin polymerization. RBOH-generated H_2_O_2_ controls localized lignin formation in Casparian strips, which improves stress tolerance [5].

NADPH oxidase, located on the plasma membrane, also plays a major role in the ROS-mediated cross-talk between cells [324]. It involves two conserved EF hand structures that can be bound by Ca^2+^ and has a potential impact on regulating oxidase activity. The transfer of electrons to O_2_ molecules and the Ca-mediated cellular cross-talk, activation or inactivation production of O_2_^•−^ is regulated by plant NOX, and the O_2_^−^ can rapidly invert to H_2_O_2_ by the enzyme SOD. Modification in the *Rboh* genes supports the development of crops that can survive oxidative stress [324].

## 8. Conclusions and Future Direction of Research

A comprehensive approach towards the signaling potential of ROS highlights the importance of different cellular events induced by stress stimulus to improve the tolerance level of plants. Manipulation of the ROS biosynthetic pathway and ROS-induced cellular events, cyclin-dependent kinases and MAPK signaling, and stress-responsive genes can enhance crop resilience. The exploitation of novel biotechnological tools for the regulation of transcription factors and signal transduction pathways support to achieve it. ROS-induced changes in metabolomics and transcriptomics have to be scrutinized and the correlation of these data with phenotypic traits is extremely important in agriculture. Unraveling the functional contributions and targeted modifications of the NADPH oxidase gene family offers promising insights for the development of high-yielding, tolerant varieties. Further research is needed to elucidate the differential responses of crops to ROS, which may facilitate the development of stress-resilient varieties and improve crop productivity under stress.

## Figures and Tables

**Table 1:** ROS-dependent responses in different crop plants under abiotic stresses.

Sl. No	Crop Plants	Type of Stress	ROS-Dependent Responses	References
1	*Oryza sativa* L.	Toxic metals (cadmium, copper, or lead) and salt	Enhanced cytosolic calcium (Ca^2+^) signaling. Blocking of Ca^2+^ signaling inhibited systemic ROS propagation and LR branching in the stress-free area. OsRBOHA and OsRBOHI active in systemic ROS signaling under asymmetric stress. Expression of *OsRBOHA* and *OsRBOHI* in roots was upregulated by Cd stress.	Xu et al. [186]
2	*Oryza sativa* L. japonica	Cd	Increased the activities of CAT, POD, SOD. The contents of non-enzymatic antioxidant substances AsA, GSH, cysteine, proline, anthocyanins and flavonoids were increased.	Wang et al. [210]
3	*Sorghum bicolor* (L.) Moench	Hg^2+^ and Cd^2+^	Enzyme activity was initially increased and then decreased. Declines in fresh weight, dry weight, and proline accumulation were also documented.	Çevık et al. [211]
4	*Capsicum annuum* L.	Flooding	Up-regulation of genes such as *mitogen-activated protein kinase 3* (*MPK3*) and *calcium-binding protein 4* (*CML4*).	Gong et al. [212]
5	*Capsicum annuum* L.	Drought stress	Increased activities of antioxidant enzymes, including superoxide dismutase, catalase, ascorbate peroxidase, and peroxidase, coinciding with the up-regulation of the expression of associated antioxidant genes.	Bulle et al. [213]
6	*Vigna umbellata* (Thunb.) Ohwi and Ohashi	Salinity and drought	Higher activity of GPOX and APX in the leaf which then decreased somewhat as the intensity of stress increased.	Atta et al. [214]
7	Wheat (*Triticum aestivum*) mutants	Salinity	Antioxidant enzymes including SOD, CAT, POX, APX and GR increased with salinity.	Karimzadeh et al. [215]
8	*Amaranthus cruentus* and *A. hypochondriacus*	Salinity	Increasing the expression of *SOS1*, *HKT1*, *NHX1*, and *DGR2* genes, which encode adaptation-related proteins under salinity stress.	Emam et al. [216]
9	*Amaranthus hypochondriacus* L.	Dehydration stress	Higher accumulation of redox-sensitive polyphenolic compounds like rutin, myricetin quercetin, gallic acid, p-hydroxy benzoic acid, vanillic acid, syringic acid, and p-coumaric acid along with the pool of monophenol, diphenol, total phenol, flavonoids, anthocyanin, and betacyanin for drought- stress-raised seedling.	Aditya et al. [217]
10	*Oryza sativa* L.	Heat stress	Excessive accumulation of reactive oxygen species (ROS) in the developing anthers leads to high temperatures (HTs), causing pollen abortion and poor floret fertility in *O. sativa*.	Zhao et al. [218]
11	*Zea mays* L.	Oxidative stress	Reactive oxygen species (ROS) burst plays a critical role in haploid induction (HI). *ZmPOD65*, which encodes a sperm-specific peroxidase, as a new gene controlling HI.	Jiang et al. [219]

## Data Availability

No new data were created or analyzed in this study. Data sharing is not applicable to this article.
